# A study protocol for a pragmatic pre-post trial to determine the feasibility and effectiveness of a novel co-designed service to support health and wellbeing of older carers of older people

**DOI:** 10.1371/journal.pone.0326363

**Published:** 2025-06-18

**Authors:** Aislinn Lalor, Susan Slatyer, Elissa Burton, Christina Bryant, Déborah Oliveira, Anjali Khushu, Natasha Brusco, Natasha Layton, Den-Ching Angel Lee, Belinda Cash, Jacqueline Allen, Lisa Licciardi, Keith D. Hill

**Affiliations:** 1 Rehabilitation Ageing and Independent Living (RAIL) Research Centre, Monash University, Melbourne, Victoria, Australia; 2 National Centre for Healthy Ageing, Monash University and Peninsula Health, Melbourne, Victoria, Australia; 3 Occupational Therapy Department, Monash University, Melbourne, Victoria, Australia; 4 School of Nursing, College of Science, Health Engineering and Education, Murdoch University, Perth, Western Australia, Australia; 5 School of Allied Health and enAble Institute, Curtin University, Perth, Western Australia, Australia; 6 Melbourne School of Psychological Sciences, The University of Melbourne, Melbourne, Victoria, Australia; 7 Faculty of Nursing, Universidad Andrés Bello, Campus Viña del Mar, Vina del Mar, Chile; 8 Millennium Institute for Care Research (MICARE), Santiago, Chile; 9 Department of Geriatrics, Frankston Hospital, The Mornington Centre, Peninsula Health, Melbourne, Victoria, Australia; 10 School of Social Work and Arts, Charles Sturt University, Albury, New South Wales, Australia; 11 School of Nursing and Midwifery, Monash University, Melbourne, Victoria, Australia; PLOS: Public Library of Science, UNITED KINGDOM OF GREAT BRITAIN AND NORTHERN IRELAND

## Abstract

Older carers (≥50 years) of older people (≥65 years) are an important sub-group of carers performing valuable roles in providing informal care but often do not have the time or give priority to supporting their own health and wellbeing. Current services supporting older carers’ health and wellbeing are fragmented and inadequate. Through previous research and co-design activity by our team, an innovative multidisciplinary Carer Health and Wellbeing Service (CHWS) has been developed. The purpose of this protocol paper is to describe the rationale for the CHWS and the methods proposed to evaluate its effectiveness and implementation outcomes. The CHWS commenced in March 2024 at Peninsula Health, Melbourne, Australia. Older carers of older people can be referred from multiple sources, including self-referral. A pre-post mixed methods study design is being utilised. Initial assessments include the Carer Support Assessment Needs Tool (CSNAT) and carer prioritisation of their needs, which guides further assessment and interventions. Assessments will occur at Service intake and 6 months later. The primary effectiveness outcome is the Preparedness for Caregiving Scale, and primary implementation outcomes are reach and adoption. Interviews of carers, referrers and staff, and a cost-utility analysis will be undertaken. The target sample size is 137 carers undertaking assessment and intervention over the 15 months data collection. Generalised linear regression will be used to compare pre- and post-continuous outcome measures. Qualitative data will be thematically analysed. Results will inform future scaling up of this innovative approach to optimising health and wellbeing of older carers of older people.

**Trial registration number:** Australian New Zealand Clinical Trials Registry (ANZCTR) – ACTRN: 12625000245493.

## Introduction

Caring for an older person with care needs is usually a progressive role, where care needs often increase in number and complexity over time [1–3]. A growing proportion of carers of older people are older persons themselves [4], many of whom have multiple and increasing health problems of their own [5,6]. These carer health problems can impact their physical and mental health and quality of life and frequently contribute to increasing care support challenges over time [2,7]. Although some informal carers of older people report positive experiences associated with their caregiving role, many report significant negative impacts to their health and wellbeing [5,8,9] that may also compromise their ability to sustain the provision of informal care longer term. Sustainability of the caregiving role and maintenance of quality of life for older carers of older people is therefore dependent on having adequate support and on being able to optimally manage their own health and wellbeing.

Much of the research about carers includes carers of all ages, with little focus specifically on older carers [10], although almost half of carers in Australia are aged 55 years or older [4]. While some generic factors affect health and wellbeing of carers of all ages, additional factors and potentially different intervention approaches may be more relevant and effective for older carers. A survey of 189 older carers of older people in Australia indicated that their quality of life was moderately reduced, with 35% indicating their own health issues were a barrier to supporting their older family members [11]. Eight in ten older carers had informed their general practitioner that they were carers, or had discussed their caring role and needs with their general practitioner, however, only 46% reported receiving advice for, or support with, their role as carers. Factors associated with better quality of life for carers included specialist clinics providing advice/assistance with the caring role [11]. While 58% attended specialist clinics with their care recipient, only 19% received specific advice or support to manage their own health and their carer role. Unpublished research from the same project [11] incorporating staff interviews at specialist geriatric out-patient services corroborated similar unmet needs for older carers of older people.

There is research evidence that single intervention approaches can improve health and wellbeing outcomes for carers (including carers of older people) [12], e.g. psychosocial programs [13,14], physical activity/exercise [15–19], occupational therapy programs [20], goal setting [21], and respite care [22]. Multi-component interventions (combining two or more approaches) have also been effective [23–25]. Despite these research findings, translation of these approaches into routine practice is currently limited and fragmented, and few of these interventions focus specifically on older carers of older people. More commonly, the focus of health professionals’ attention, and the person who is usually interacting with health services for assessment and intervention, is the care recipient (not the carer). There is little research evidence detailing systematic and dedicated assessment of the needs and supports to enhance health and wellbeing of older carers of older people, or implementation research evaluating translation of this research into practice.

One report (an unpublished Master’s thesis) is the only literature identified that reports a specialist service targeting older carers of older people [26]. This Carer Clinic model operates in Sao Paulo, Brazil, and targets carers aged ≥55 years of age. The clinic’s multidisciplinary team consists of a social worker, psychologist and physician, who provide multi-disciplinary assessment and interventions, focussing on medical, social and psychosocial aspects of carers’ needs. No formal evaluation of the outcomes of this novel service has been published.

There is a need for a systematic approach to identifying and supporting older carers of older people to optimise their own health and wellbeing while sustaining their caregiving role [27]. We have undertaken recent research activity with key stakeholders, that included voices of older carers, carer support organisations, health professionals, health care organisation administrators, policymakers, and researchers with expertise in health and wellbeing of older carers, to co-design the development of a novel Carer Health and Wellbeing Service (CHWS) in Melbourne, Australia [28]. Key elements of the model of care determined through the co-design panels included being: (1) driven by carer-identified needs and priorities; (2) interdisciplinary, with flexibility in initial screening and subsequent tailoring of assessments and interventions to ensure relevance to individual carers (including referral to other services if required); and (3) able to provide multiple modes of service delivery and resources, including face-to-face, telehealth (supporting those with difficulty leaving home, or in regional/rural areas) and website resources [28].

This protocol paper details the methods being used in the implementation of the new CHWS and evaluation of the impact of the service on the health and wellbeing of older carers (for this study, defined as carers aged 50 years or older) of older people (defined as people receiving informal care aged 65 years or older). This study aims to evaluate (1) the effectiveness outcomes for older carers participating in the CHWS; (2) the implementation outcomes associated with the CHWS – including feasibility, reach, acceptability (carers, Service staff, referrers), maintenance and fidelity; and (3) the cost-utility of the CHWS.

## Methods

**Fig 1:**
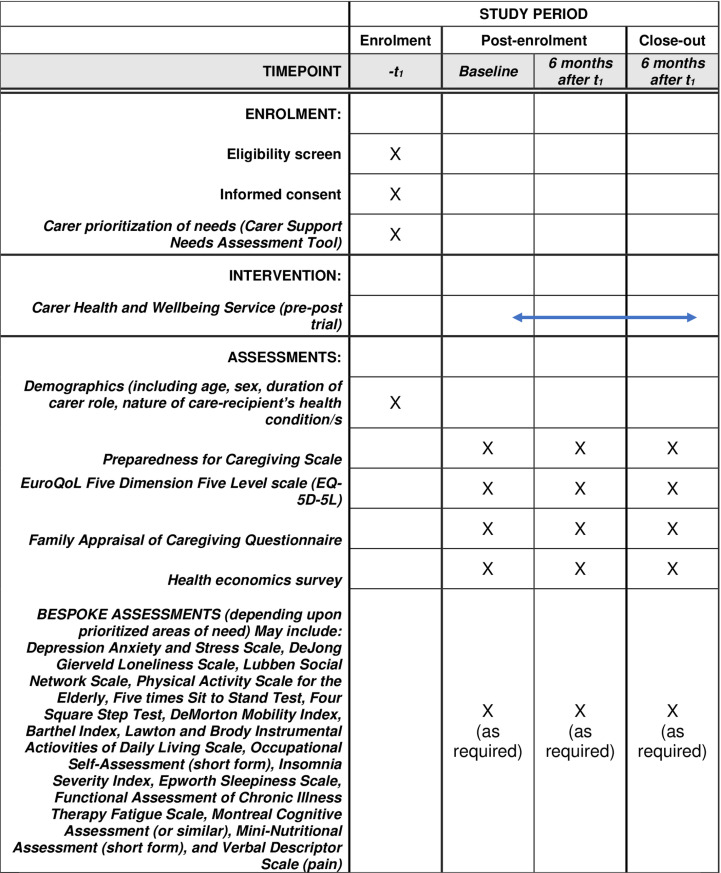
SPIRIT Checklist for schedule of enrolment, interventions and assessments.

A mixed methods one-group pre-post (6-month) study design will be utilised, using both quantitative and qualitative data. The protocol follows the checklist and guidelines described in the SPIRIT guidelines for the content of clinical trial protocols (Fig 1, and Supporting Information S1 Checklist) [29] and the ‘Template for Intervention Description and Replication (TIDieR) (Supporting Information, S2 Checklist) [30]. The project commenced recruitment on March 1 2024, recruitment will end on 31 May 2025, and the project will end in December 2025. The study was retrospectively registered in the Australian New Zealand Clinical Trials Registry (ANZCTR; number ACTRN: 12625000245493). The delay in study registration, which occurred after the commencement of participant enrolment, was primarily associated with the complexities of establishing a new service within a health service and its requirements; and a six week delay in early 2025 when the trial registration site was closed due to a cyber security incident. The authors confirm that all ongoing and related trials for this intervention are registered.

### Setting

The CHWS commenced operating on March 1 2024 from a community centre in Frankston (Melbourne, Australia) as an outpatient service, with opportunities for in-person and/or online assessments and interventions. During the establishment of the CHWS, staff completed a training program that included: (1) an overview of the CHWS operations and procedures (described below); (2) induction training required for new staff at Peninsula Health (the health service through which the CHWS was established); (3) training regarding all CHWS assessment procedures. One of the primary tools being used to guide discussion and prioritisation of carer needs at the initial CHWS assessment is the Carer Support Assessment Needs Tool (CSNAT) [31]. Staff have undertaken the required online training program for the use of the CSNAT before the Service commenced operations (https://csnat.org/). Licencing permission for use of the CSNAT, according to the authors’ defined terms of use has been received for this project (https://csnat.org/licensing/); (4) training for staff to support capacity building for carers in problem-solving for current and future potential problems associated with the carer role (based on a training program for a previous intervention trial for carers of older people being discharged from hospital) [32]; and (5) research data management. The training program included face-to-face (1–2 days) and online components.

### Staff governance

CHWS staff are employed through Peninsula Health. Operational governance (e.g., induction and orientation to employment, payroll, etc) is being overseen by Peninsula Health. Discipline-specific clinical supervision for each of the CHWS clinicians is provided through Peninsula Health staff and their existing supervision support pathways. Research and other clinical governance (e.g., training associated with various assessment tools, research requirements of the Carer Service, research skills, etc) are being overseen by the research team at Monash University.

### Ethics

The project has received approval from the Peninsula Health Research Ethics Committee (LNR99736PH-2023) and reciprocal ethical approval from the Monash University Health Research Ethics Committee (2023-40977-100362).

### Participants and recruitment

Participants include (1) older carers of older people (termed “carers” from this point); (2) referrers to CHWS; and (3) CHWS staff.

#### Carers.

Carers are defined as “family members and/or friends who routinely support the older person through assistance with household tasks; self-care and mobility; emotional and social support; treatments, medication and responding to acute health needs; advocacy and care coordination; or surrogate decision-making” [33]. The inclusion criteria for carers attending the CHWS, and for this study are (a) being aged ≥50 years; (b) living in the community; and (c) being an informal (unpaid) carer (as defined above) for a person aged ≥65 years living in the community.

Carers can be referred to the CHWS through referrals (e.g., general practitioners, allied health professionals, geriatrics services and specialist clinics, community health centres, and community aged care organisations) or be self-referred (e.g., through hearing about the CHWS from peers, media, or other avenues of service promotion). During initial contact with the CHWS, carers will be asked if they are willing to provide written informed consent to participate in the research evaluation of the CHWS. Whether or not the carer consents to participate in the research study, all carers will receive the same service provision through the CHWS. However, only those consenting to participate in the research will have their data included in the evaluation of the service.

During an initial phone contact, a CHWS staff member will complete a screening demographic questionnaire with the carer to ensure they meet the study inclusion criteria. Once eligibility and interest in participation in the study are determined, the carer will be asked to review the explanatory statement and consent form, and if willing to participate in the research, to sign the consent form and return it to the CHWS staff. CHWS staff will then inform the research team of carers who have agreed to participate in the research. Involvement in the research study will include allowing data from baseline and 6 month follow-up assessments to be used to evaluate the service outcomes and possibly being asked to be involved in a semi-structured interview after the 6 month assessment (which will involve a separate explanatory and consent form). Participants will be purposefully sampled for these interviews, aiming for diversity of age, years in the carer role, nature of prioritised needs, and health status of the carer. Carers who would like to find out more about the study before consenting to participate will be able to follow up with the CHWS staff member, or members of the research team.

#### CHWS staff.

Staffing of the CHWS includes a social worker, a psychologist, a physiotherapist, and an occupational therapist, all with experience working with older people, and their carers. Initial staffing for the clinical psychologist and the social worker will be one day/week; while the physiotherapist and occupational therapist will work 1/2 day/week. Staffing levels (time) will be doubled in the second year of the CHWS operations (2025). In the last three months of recruitment, all CHWS staff will be invited to participate in an interview as part of the service evaluation. Staff will be informed that participation in the interview will be voluntary and that their decision to participate in the interview or not will not affect their employment with the CHWS. Staff willing to be interviewed will provide written informed consent. As part of ensuring the new Service is responsive to issues identified as it becomes more established, there will also be opportunities for meetings between the researchers and the CHWS staff intermittently to discuss CHWS operations and potential areas for clarifying or modifying aspects of the Service.

#### Health professionals who referred carers to the CHWS.

Any health professional providing two or more referrals to the CHWS (e.g., general practitioner, allied health professionals, geriatrics services and specialist clinics) will be invited to participate in the study evaluation through a semi-structured interview. Invitations to participate in the interviews will be sent through the CHWS manager to referrers, who will be asked to contact a member of the research team if interested in being involved. Referrers will be asked to provide written informed consent to take part in the interview.

### Procedure: Service screening and assessment tools

#### Older carers (n=137).

**Fig 2:**
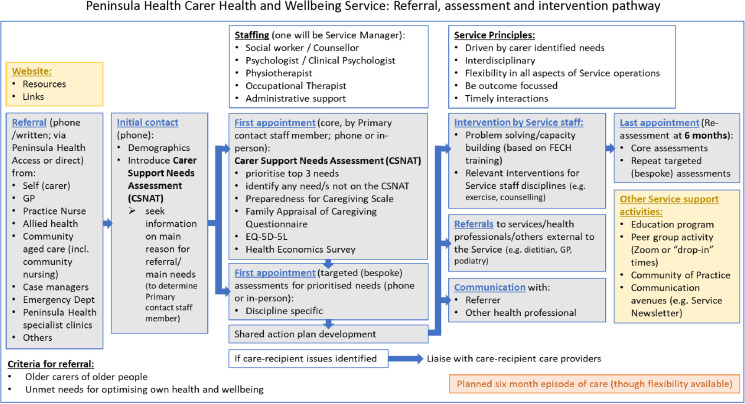
Referral, assessment and intervention processes for the Carers Health and Wellbeing Service (CHWS).

An episode of care for carers attending the CHWS is up to 6 months in duration, although this may vary depending on individual carer needs. Fig 2 shows the referral, assessment and intervention flow for carers referred to the CHWS. Referrals will be received by the CHWS manager or administrative support person, using a standard intake referral form (Access form) either through the Peninsula Health Access Manager (the usual process for referral intake/processing at Peninsula Health), or direct from the referrer to the CHWS administrative person. The carer will then be contacted by the CHWS manager or administrative support person to undergo orientation to the CHWS and associated study, involving six steps:

a) Description of CHWS purpose and processes (if not provided by the referrer to the carer);
b) Screening demographic questions and questions to ensure the carer meets the inclusion criteria for participating in the CHWS;
c) Description of the option for participation in the research study and their participant data being used as part of the CHWS evaluation (and the need for signed informed consent for this to occur) (Note: consent to participate in the research is not a prerequisite to use the CHWS services, so those not providing consent for the research will still work through all the CHWS processes, but the research team will not have access to their data);
d) Introduction to the CSNAT, and request for the carer to review and reflect on the CSNAT questions in preparation for the initial CHWS assessment (which will be the same process whether it is conducted face-to-face or online);
e) Carers’ initial perception of the main area/s they are seeking assistance, information, advice or interventions to assist them to maintain or improve their own health and wellbeing (to guide decision-making by CHWS staff regarding the most appropriate team member (discipline) to conduct the initial assessment – this will be accommodated as best as possible based on information provided and availability of the relevant staff member). Adjustments may be made in terms of the CHWS staff member to undertake the initial assessment to minimise delays in commencement with the services if required; and
f) Preferred mode of Service participation (in-person or online), and preferred mode for receiving information from the Service (email, phone, post).

The carer’s first appointment will be scheduled where possible with the CHWS staff member whose professional expertise best aligns with the primary needs identified by the carer during screening. This staff member will be designated the carer’s key contact person, who will assume overall primary contact and oversight of the carer’s CHWS management. The first appointment will have a focus on: completing all core assessments (Table 1); identifying prioritised carer needs (up to three top prioritised needs, based on the carer views in the context of the CSNAT and any other needs); and shared decision-making regarding initial area/s to address and intervention/s (what matters most).

**Table 1:** Core assessments (used for all CHWS clients) and bespoke assessments (used depending upon prioritised needs being addressed for individual clients).

Measure^*^	Description
**CORE ASSESSMENTS**	
Carer Support Needs Assessment Tool (CSNAT) [31,35]^*^.	The CSNAT is a 14-item tool (seven domains of support enabling the carer to care, and seven domains of support relating to their own wellbeing), used to obtain information on and prioritise carer’s support needs. It can be used as both a research tool and as part of a practice intervention (staff using the CSNAT will undergo required training). *The CSNAT will not be used as an outcome measure, as recommended by the tool developers.*
Preparedness for Caregiving Scale (PCS) [34]^*^.	**Primary outcome measure.** The PCS includes eight items evaluating how prepared carers consider themselves to be for different aspects of caregiving. Each item is rated on a five-point scale (0 = not at all prepared; 4 = very well prepared). Higher scores indicate better preparedness for caregiving. Components of the PCS and overall PCS score have been shown to be responsive to change in a nurse-led intervention for family carers of older people being discharged from hospital [36].
EuroQoL Five Dimension Five Level scale (EQ-5D-5L) [37]^*^.	The EQ-5D-5L is a widely utilised measure of quality of life that is also used in health economic evaluations. For this study, the five domains of the EQ-5D-5L (mobility, self-care, usual activities, pain/discomfort and anxiety/depression) will be assessed, each on five levels (no problems, slight problems, moderate problems, severe problems and extreme problems).
Family Appraisal of Caregiving Questionnaire (FACQ) [38]^*^.	The FACQ has 26 items, rated on a 5-point scale ranging from 1 (strongly disagree) to 5 (strongly agree). The 26 items are grouped into four subscales, including: caregiver strain, positive caregiving appraisals, caregiver distress, and family wellbeing.
Health economics survey to capture care related data and health care utilisation data including emergency department and hospital admissions over the past 6 months^*^	This survey will ask average weekly hours of care provided by the carer, by other people who are not paid, and by other people who are paid; as well as capture the “preferred” and “actual” weekly hours the carer spends on their top three leisure activities (where the carer role contributes to the gap between “preferred” and “actual” hours of participation). It will also include self-report health care utilisation information. This information will be used in the cost utility analysis.
**BESPOKE ASSESSMENTS** ^**^	
**Psychological health and wellbeing**	
Depression, Anxiety and Stress: The 21-item (short form) Depression, Anxiety and Stress Scale (DASS-21) [39]^*^.	This self-report scale measures the negative emotional states of depression, anxiety and stress, and has been shown to have sound psychometric properties with older people [44]. Given the tool is designed to measure a number of constructs, this reduces the need for multiple scales across these constructs, thereby reducing potential burden for participants.
Loneliness: The De Jong Gierveld 6-item Loneliness Scale [40]^*^.	The six items are scored yes, more or less, or no and focus on emotional and social loneliness.
Social participation: the abbreviated Lubben Social Network Scale (LSNS-6) [41]^*^.	The LSNS-6 consists of three items in each of a Family domain and a Friendship domain. Each item is rated on a 0–5 rating scale.
Psychosocial assessment ^*^	An in-depth discussion to understand the experiences of the caregiver both as an individual and within the context of their family and community. This type of assessment is used to identify potential strengths and resources available to caregivers, as well as possible challenges within their social context that could be addressed to improve health and wellbeing.
**Physical performance**	
Level of physical activity: The Physical Activity Scale for the Elderly (PASE) [42]^*^.	The PACE includes evaluation of leisure, household and occupational activity, that can be combined (utilising frequency, duration and intensity level of the physical activity) to derive an indicator of the level of light, moderate and vigorous physical activity.
Lower limb muscle strength: using the Five Times Sit-to-Stand Test [43].	Participants will be asked to sit on a chair (43 cm high), stand up (with arms crossed over their chest if possible) and sit back down five times as quickly as possible. The time taken to perform five complete sit-to-stands will be measured (seconds). If unable to stand up with arms crossed over their chest, participants will be asked to perform the task allowing pushing up with their arms (with use of arms documented). Performance will be timed in the same way if arms are used.
Dynamic balance: Four Square Step Test – a dynamic rapid weight shifting and stepping in multiple directions task [44].	Four walking canes (or similar) are set up in a + shape on the floor; the participant stands in the left forward quadrant facing forward. When instructed to start, they step as quickly as possible for both feet to be positioned in the right forward quadrant, then backwards for both feet to be in the right backwards quadrant, then to the left to be in the left backwards quadrant, then forwards to the starting quadrant; and then reverses the stepping procedure. Timing of the task stops when the person returns to starting position again (i.e., has stepped in 4 quadrants clockwise and four quadrants anticlockwise).
Balance and mobility: The de Morton Mobility Index (DEMMI) [45].	The DEMMI is a 15-item test of balance and mobility. Items are hierarchically ordered, so if a person is unable to perform lower-ordered items, they will not be able to do higher-ordered items. Scores will be converted to a RASCH analysed score for analyses [41].
**Function**	
Activities of Daily Living (ADL): the Barthel Index [46]^*^.	The Barthel Index is widely used to assess functional independence. It uses an ordinal scale across 10 domains of ADL or mobility activities.
Instrumental ADL (IADL): the Lawton and Brody Instrumental Activities of Daily Living Scale [47]^*^.	This eight-item tool assesses activities such as shopping, the ability to handle finances, and handling own medicines. Each item has between three to five response options, each scored as 0 or 1.
Functional competence: the 12-item Occupational Self-Assessment-Short Form (OSA-SF) [48]^*^.	The OSA-SF enables self-reporting of perceived competence across areas of performance/participation and the level of importance of the activity for the individual.
Sleep quality/disturbance will be assessed through the Insomnia Severity Index (ISI) [49]^*^ and the Epworth Sleepiness Scale (ESS), respectively [50]^*^.	The ISI is a 7-item screen (with a 5-point Likert scale) to assess the nature, severity and impact of insomnia. The ESS is an 8-item self-administered questionnaire where respondents rate, between 0–3 their chance (from never to high chance) of dozing off during 8 activities. Both tools are brief and have strong psychometrics.
Fatigue: The 13-item Functional Assessment of Chronic Illness Therapy (FACIT) Fatigue Scale [51]^*^.	The FACIT Fatigue Scale is measured on a 4-point Likert scale and is specifically designed to assess fatigue in older adults.
**Other measures**	
Cognition	The Montreal Cognitive Assessment (MoCA) (ST) [52]; Rowland Universal Dementia Assessment Scale (RUDAS) [53]; Trail Making Test (TMT) (A) [54].
Nutrition^*^	The Mini Nutritional Assessment-Short Form (MNA-SF) (ST) [55].
Pain^*^	Verbal Descriptor Scale (VDS) (ST) [56].

* Within each assessment domain, at least one of the assessment tools will be able to be undertaken remotely, or is a questionnaire, to enable some outcomes to be evaluated for carers who are only able to attend the CHWS remotely. These tools are denoted with an asterisk.

** Bespoke assessment tools will be selected upon need, as determined by the CHWS staff member, based on prioritised area/s of need by the carer.

Assessment tools for carers, where applicable, will be utilised both as outcome measures and to inform possible interventions (e.g., data from the physical performance assessments may guide the type of exercises to include in a home exercise or physical activity program) (Table 1). Assessment tools are listed in Table 1 as (1) Core assessment tools, to be administered to all carers on initial assessment; and (2) Bespoke assessment tools, listed under the domains of psychological health and wellbeing, physical performance, function, and other assessments. One or more bespoke assessment tools will be utilised depending on the domains or areas of need prioritised by carers to be addressed through the CHWS intervention. The primary effectiveness outcome measure will be the Preparedness for Caregiving Scale [34]. All assessments may be performed by any of the CHWS staff (who will all be trained in their use) although it is anticipated in most cases the physical performance assessment will be undertaken by the physiotherapist, functional assessments by the occupational therapist, and psychological health and wellbeing assessments by the psychologist or social worker. For each domain, at least one of the assessment tools will be able to be undertaken remotely, or be a questionnaire, to enable some outcomes to be evaluated for carers who are only able to attend the CHWS remotely. Although the CSNAT data will be recorded to document carer-prioritised needs at baseline, at the 6 months assessment it will only be used to ask carers if the initially prioritised needs have been (1) fully addressed; (2) partially addressed/ still being worked on; or (3) unchanged (still remains a major prioritised need).

Assessments will occur at initial intake assessment, and 6 months later, as the Service will operate typically with a 6 month episode of care. However, for carers who feel their needs have been fully met earlier than 6 months, the final assessment will be brought forward. For carers who may still have active issue/s being worked on with staff, a 6 month assessment will be completed (to be used in main data analysis), and a final assessment undertaken at a later time point when the episode of care is completed.

Depending on the identified carer prioritised needs and shared decision-making, the CHWS staff member (or another staff member if more relevant) will undertake one or more additional bespoke assessments related to the area/s of need identified. For example, if the areas of need related to depression and poor sleep, additional assessments could include the 21-item (short form) Depression, Anxiety and Stress Scale (DASS-21) [39,57], the Insomnia Severity Index (ISI) [49], and the Epworth Sleepiness Scale (ESS) [50]. Table 1 lists the main bespoke assessment tools that will be utilised depending upon the prioritised needs of the carer. Additional assessment tools may be selected by the CHWS staff member if a domain of interest is not covered by the bespoke tools listed in Table 1.

As well as the core and bespoke (individualised) assessment tools used for each carer, the Client Satisfaction Questionnaire (8-item, CSQ-8) will be completed by all carers [58]. Additional client satisfaction questions will evaluate carer perspectives of the CHWS referral processes, carer needs prioritisation processes, assessments, interventions, interactions and communications with CHWS staff, modality of interactions with CHWS (face to face or online, or hybrid/ mix), referrals from CHWS and communications with other relevant medical or health professionals, access to relevant information and resources, timeliness of processes and communications, and what they considered worked well and what aspects did not work well. The CSQ-8 and additional service satisfaction questions will be available online (through Qualtrics) or in hard copy. The additional satisfaction questions will be rated using a 7-point Likert scale response, as well as some open-ended questions.

A purposeful sample of approximately 15–25 carers completing the 6 month assessment will also be asked if they are willing to participate in a semi-structured interview (estimated duration 45 minutes). The sample size for interviews will be determined by data saturation. Interviews will be conducted by an experienced qualitative researcher face-to-face or online, dependent upon the preference of the carer, and will be recorded to ensure the accuracy of reporting and analysis. The interviews will explore in more depth the questions in the CHWS satisfaction survey, as well as seek perspectives about recommendations on how the CHWS could be improved. Carers who participate in the interviews will be provided with a $AUD50 gift card as an acknowledgement of their time contribution.

#### CHWS staff (n = 4).

Staff working at the CHWS within the last six months of the evaluation of the CHWS will be sent a letter (via email) by a member of the research team, inviting them to participate in the evaluation of the CHWS. Consenting staff will be involved in a semi-structured interview (approx. 30–45 minutes) focused on their perspectives about, and their acceptability of the Service over the previous 18 months. Location/mode and timing of the interview will be determined in discussion with consenting staff and can be conducted during their funded work time with the CHWS. Interviews will be conducted by an experienced qualitative researcher and will be recorded to ensure the accuracy of reporting and analysis. If any staff leave the CHWS during the two-year evaluation, they will be asked to undertake an exit interview (similar questions as described above).

#### Referrers to the CHWS (n=20–25).

A purposeful sample of referrers to the CHWS who provided two or more referrals to the CHWS will be sent a letter from the CHWS manager (via email), inviting them to participate in the evaluation of the CHWS, together with an explanatory statement and consent form. Purposeful sampling will aim to achieve diversity in the disciplines of referrers (e.g., general practitioners, and allied health professionals). Referrers interested in participating will be able to contact the research team to complete their consent to participate. Consenting referrers will undertake a semi-structured interview (approx. 30 minutes) focused on their perspectives about, and their acceptability of, the CHWS based on their knowledge and interactions with the service, and the clients they referred to the CHWS. The location/mode and timing of the interview will be determined in discussion with consenting referrers. The sample size of referrers will be determined by data saturation. As an acknowledgment of their time, referrers will be provided with a $AUD50 gift card. Interviews will be conducted by an experienced qualitative researcher and will be recorded to ensure the accuracy of reporting and analysis.

#### Implementation (service-related) outcomes.

As well as the carer-related outcomes detailed and in Table 1, the research team will also be evaluating **implementation outcomes** for the CHWS (how well the CHWS succeeds in meeting key service-related implementation outcomes). These will be based on the RE-AIM framework (components are Reach, Effectiveness, Adoption, Implementation, and Maintenance) [59]. Primary implementation outcomes are reach (outlined below) and adoption (% completing intervention and 6 month assessments).

a) *Reach* will be determined through referrals received, aiming for >80% of appointment slots for all CHWS staff being utilised in the last six months of the study recruitment period; and overall sample size relative to the planned sample size (see analysis and sample size calculation section). Sources of referrals (medical practitioners, allied health professionals, carers themselves) will be reported;
b) *Effectiveness* outcomes will mainly include the carer outcome measures (Table 1), and also a *cost-effectiveness* evaluation. A cost-utility analysis will be conducted using a societal perspective over a 12 month time horizon (6 months before the carer’s first appointment and 6 months following the carer’s first appointment). Cost data will include CHWS intervention costs (excluding research costs); carer emergency department presentations and hospital admissions (self-report); the quantity of care provided to the person requiring care, including average weekly hours of care provided by the carer, by other people who are not paid, and by other people who are paid (self-report). In addition, the carer will report the “preferred” and “actual” weekly hours spent on their top three leisure activities (noting when the carer role contributes to the gap between “preferred” and “actual” hours of participation). Effect data will be based on the utility index derived from the EQ-5D-5L [37], measured during the carer’s initial appointment and again at their 6 month follow-up assessment. The change in utility index between initial assessment and 6 months will be used to determine quality-adjusted life years (QALYs) gained or lost by the carer. Cost and effect data will be combined to determine incremental cost-effectiveness ratio (ICER) per QALY gained, with a threshold of <$AUD50,000/QALY gained to determine cost-effectiveness [55].
c) *Adoption* will be evaluated through several service system outcomes (percentage completing intervention programs and 6 month assessment, number of interventions undertaken, and number of intervention sessions undertaken with CHWS staff). Acceptability of the service will be evaluated through the CAQ-8 and associated survey items, and interviews of a purposive sampling of carers (after the 6 month assessment), service referrers, and an interview with consenting CHWS staff in the last six months of the intervention, or an exit interview for staff leaving the service;
d) *Implementation* includes fidelity of the program as intended (which will be evaluated by members of the research team reviewing 5% of de-identified patients’ assessments and intervention programs implemented against CHWS protocol) and adaptations (CHWS staff will document issues and adaptations made during the CHWS implementation and report these to the research team; and will be asked to comment on adaptations in the staff interviews). Other service-related aspects of implementation will be captured as part of the economic evaluation (e.g., costs, non-attendance at booked sessions);
e) *Maintenance* at the individual (carer) level will be evaluated through the proportion of carers completing the 6 month assessment and completing interventions. At the system level, maintenance will be determined through the CHWS being able to continue beyond its initial two year intervention period.

### Procedure: Interventions for carers

Interventions will be determined through shared decision-making between the carer and their primary contact CHWS staff member and will be informed by the carer-prioritised needs (CSNAT and other needs), and assessment findings. Depending on the expertise and capacity of CHWS staff, some interventions will be delivered by CHWS staff, while other interventions may require referral to other practitioners or services, within or external to Peninsula Health. Carers will be encouraged to continue other health and wellbeing strategies they may be undertaking at the time of attending the CHWS.

#### Interventions provided by the CHWS or referrals to other practitioners or services.

Following intake assessments at the CHWS, an initial individualised intervention plan will be developed with the carer. This plan will be reviewed regularly to ensure interventions are adapted if necessary to meet changing needs. Interventions will be individualised to the carer and may be changed over time through ongoing shared decision-making between the carer and the CHWS staff, depending on progress and possibly changing needs. The CHWS staff will follow up with the carer to support implementation of one or more intervention options that are within their scope of practice to deliver, through provision of information or resources, through direct intervention provision (e.g., if the Primary Contact is a physiotherapist, and the carer has strength or fitness needs, they may be provided with a tailored home exercise program or participation in a CHWS-based group exercise program), or through other actions as required.

There will be broad flexibility in the type and nature of intervention/s that will be initiated in the first instance, including whether the intervention is delivered by CHWS staff, whether it involves referral to an external service/provider/practitioner, and its mode of delivery (face-to-face, online, or hybrid). If assessments indicate the need for medical review for the carer, they will be informed of this, and with their consent, provided with a letter to their medical practitioner outlining assessment findings, planned interventions, and reasons for recommending a medical review. Information on possible options for interventions are included in Table 2, although this is not an exhaustive list.

**Table 2:** Examples of intervention options for to be provided to carers by CHWS staff, or through referral to other practitioners of services.

Discipline	Services that may be delivered through CHWS staff	Services that carers may be referred outside of the CHWS for^*^
**Social worker**	Counselling or other therapeutic interventions to explore identified issues; Facilitation of individual or group work sessions focused on relevant topics, such as: strategies to navigate changing relationships, self-care and wellbeing, care planning and accessing supports, advocating for themselves and their care recipient, and the importance of maintaining social connections.	Specialist counselling or therapeutic supports (e.g., to support issues including those relating to family violence, family therapy, mental health, or financial counselling); Group or social activities to support social connection and wellbeing.
**Clinical psychologist**	Brief interventions for anxiety and depression; Psychoeducation regarding the caring role; Interventions to support self-care.	Bereavement counselling; Relationship counselling; Medium to long-term therapy for long-standing concerns, e.g., past trauma.
**Occupational Therapist**	Relaxation, mindfulness and stress management techniques; Energy conservation techniques and associated carer education (e.g., 4Ps: planning, pacing, prioritising, and positioning); Home assessment (where possible) to identify risks and or improve carer health and wellbeing as they perform their carer role (e.g., correct use of equipment in the home); Training for manual handling; Assistive technology to support the carer at home.	Carer respite Specialist sleep intervention
**Physiotherapist**	Exercise options, including provision of (1) a tailored home exercise program; (2) a group exercise program (if CHWS physiotherapist has time capacity and relevant experience to deliver); (3) individual 1:1 physiotherapist supervised exercise program (in limited circumstances, depending on time capacity); or (4) other advice regarding maintaining/improving physical performance; Physical activity advice and support/ motivational interviewing (including opportunities for physical activity with care recipient; or independent of the care recipient); Assistance or advice regarding manual handling including for managing the care recipient, and handling of aids and equipment; • Assessment and advice regarding joint pain; • Assessment and advice regarding the need and use of mobility/gait aids (if required for the carer).	Exercise options not able to be provided through the CHWS, including referral and provision of details of a group exercise program (e.g., tai chi, hydrotherapy) not available through the CHWS physiotherapist, or when unable to attend; • Complex pain management, manual therapy, or pain management options not available through the CHWS (e.g., electrotherapy).
**Other**		**Referrals to external organisations for further assessment or interventions:** Where carers’ needs are not able to be addressed within the CHWS (e.g., outside of scope for the CHWS staff, delay in commencing intervention due to wait-list (if present)), carers will be offered referral to a suitable service or provider external to the CHWS, so that the intervention can be implemented. Examples may include referral to: a geriatrician, a general practitioner or a pharmacist for medical or medication-related issues or review; a dietitian for the management of dietary or nutritional issues; a sleep studies service for further assessment and management of sleep difficulties; or a podiatrist for management of foot pain or other foot problems.

* Data regarding these referrals will be recorded in the carers’ Electronic Medical Records and will be collected as part of the evaluation of the CHWS.

#### Other interventions provided through the CHWS (in addition to those outlined above and in Table 2).

Staff will have a range of publicly available information resources to provide to carers, or for carers to search through a structured online portal. While much of the information resources will be focused on carer health and wellbeing, some are also related to improving the carers’ understanding of the care recipient’s specific health condition/s, possible prognosis, and what to expect and prepare for. Where indicated, carers may be referred to their or their care recipient’s medical practitioner for further details of the care recipient’s status and prognosis.

The CHWS staff will run intermittent education sessions to support carers (may be online, face-to-face, or hybrid), and/or link carers to other existing programs available within the community/other organisations, to address commonly identified issues that may benefit from a group education approach. Opportunities for carer peer support (through face-to-face or online) will be provided.

#### CHWS staff team discussion and communication with medical practitioners and other service providers.

After a carer’s initial appointment or 6 month assessment, and at other times as required, the CHWS team will meet to discuss identified needs, intervention plan, outcome/s and other relevant issues. On these occasions, a letter from the CHWS Primary staff contact will be sent to the carer’s general practitioner (or other service provider, as recommended by the carer) outlining the CHWS staff assessment findings, intervention plan/s, and after the 6 month assessment – outcomes of the intervention and ongoing plans.

#### Subsequent appointments and episode of care.

CHWS staff may schedule face-to-face, phone or online appointments with carers to follow up on aspects of interventions or activities after the first appointment, at a frequency that is mutually agreed upon between the carer and the staff member.

Carer involvement with the CHWS will last an average of 6 months (episode of care), from initial contact to final assessment, **with a standard episode of care being six months duration**, from the time of completion of the initial assessments. The decision regarding discharge timing will be made by the CHWS Primary contact staff member and the carer.

Although it is anticipated that the standard episode of care will be six months, carers with ongoing needs being actively addressed by the CHWS or who have added one or more extra interventions during the 6 month period may opt to continue with the Service beyond the 6 month mark. Those who complete an episode of care with the CHWS can be re-referred if their circumstances change, or new issues arise.

For situations where the person being cared for transitions from home to residential care (for permanent care, not for respite care), or in situations where a care-recipient passes away during the episode of care for the carer, the carer will have the option to continue with CHWS support (with review to determine if needs have changed), or to cease involvement with the CHWS (with re-assessment at this time point if this is acceptable to the carer).

The level of risk for adverse events associated with participating in the CHWS is considered low. If an adverse event occurs, it will be reported to the CHWS Manager, and then to the research team, who will inform the Ethics Committee. The health professional/s involved will provide or organise short term assistance and care as required, including supporting seeking medical support.

### Data collection and statistical analyses

Comprehensive data collection will be established via the Peninsula Health Electronic Medical Records (EMR), including participant profile data, the Carer Support Needs Assessment Tool, core outcomes, and main assessments. Data will be entered by Service staff following each assessment, and will be stored securely on the Peninsula Health site. The UR (unique identifier) for carers who have consented for their data to be accessed to aid evaluation of the CHWS, will be provided to the Peninsula Health ACCESS (intake) team. Members of the ACCESS team will access the relevant assessment and intervention data for participating carers, and will extract de-identified data for carers who consented to participate in the evaluation. Data analysis will be conducted using SPSS. Data will be stored securely on the Peninsula Health password-protected site, and de-identified data for the research team analyses will be stored on the Monash University secure site.

### Sample size calculation

This is a pragmatic evaluation study of a new service at Peninsula Health, which will recruit for a 15 month period, with six month follow-up assessments. In the first 10 months of operation, the CHWS will operate one day/week, and for the subsequent five months, two days/week (to allow time for growth in referral base). Conservatively, we estimate two new referrals/ week on average for the first 5 months of CHWS operations, 4/week for months 6–10, and then for the final five months of recruitment (with the CHWS operating 2 days/week) 8 referrals/week. Based on 48 weeks/year of operation, over the 1.25 years of recruitment for the study, this will mean an anticipated 248 people referred. We estimate that 55% of carers referred to attend the Service will consent to participating in the project (n = 137) and undergo assessment and intervention. A previous intervention study to support carers of older people being discharged home from hospital achieved a significant mean improvement of 0.2 on the total average item score for the primary effectiveness outcome measure in this study – the Preparedness for Caregiving Scale [34] (mean average item score at baseline = 2.67 (0.57 sd) on the 0–4 scale for each item, with a sample of n = 140 (two groups)) [36]. This sample size is also expected to be sufficient for the majority of outcome measures being collected.

### Data analysis

Extracted data for the CHWS evaluation comparing baseline and 6 month assessment data will be analysed using parametric analyses for continuous, normally distributed data, and non-parametric analyses for categorical or non-normally distributed data. Analyses for comparison of the continuous core and additional domain assessment between initial and 6 month assessments will be performed using generalised linear regression. Analyses will be conducted on an intention-to-treat basis.

Analysis for the cost effectiveness evaluation will involve the costing of items based on actual costs where available, and where these are not available, costs, excluding carer time, will be based on market rates, with carer time based on the minimum wage. All costs will be presented as AUD 2025/26, with costs collected prior to 2025/26 to be inflated by CPI [60]. The EQ-5D-5L raw scores will be converted to a utility index using Australian weights [61], and then into QALYs. The ICER will be calculated using the bootstrap method (5,000 replications) with the results presented on a cost-effectiveness plane and as a probability of cost-effectiveness across a range of willingness to pay thresholds (AUD $0 to $50,000) [62].

For the qualitative study (semi-structured interviews) of carers and referrers, recruitment will continue until data saturation for the carers and the referrers groups. Thematic analyses will be undertaken independently by two members of the research team for each participant group, using Braun and Clarke’s six stages of reflexive thematic analysis [63]. In cases of disagreement between the coders, the two team members will discuss their perspectives to achieve agreement on themes. The thematic analyses will then be presented to a subgroup of the research team and the CHWS staff (n = 6) to review and discuss themes, for finalisation of the thematic analysis for each participant group. A similar approach will be used for analysis of the CHWS staff interviews, although the number of interviews will be small and constrained by the number of current staff, together with interviews with any staff who leave the service. Commonalities and differences in themes between the participant groups will be identified. All quotes to support themes will be de-identified.

### Dissemination of study findings

A plain language summary (de-identified) will be provided on the Rehabilitation, Ageing and Independent Living (RAIL) Research Centre website and forwarded to all participants at the end of the project. Study results will be disseminated through conference presentations and publications.

## Discussion

Older carers of older people represent a large and important subgroup of carers, who lack systematic approaches and support to understand and manage their own prioritised health and wellbeing needs [1,3,5,64–67]. If their health and wellbeing are not able to be maintained at a sufficient level, there is a high risk of them not being able to sustain their valuable caregiving role [3]. While there are a growing number of services and supports for carers of all ages, and providing care to people of all ages, the majority focus on facilitating service system navigation (finding appropriate services and supports) and information resources. However, rarely are there opportunities for a multidisciplinary assessment of the health and wellbeing of the carer [27], which is the focus of this novel CHWS.

While older carers of older people may have interactions with geriatric services and primary care for the care of the person they provide care for, these primarily focus on health assessments of the person receiving care [66], with carers often feeling forgotten and not considered an integral component of the care team to enable sustained independence of the person they provide care for [68]. An Australian survey of older carers of older people indicated that of those attending specialist outpatient geriatric services, less than 20% of carers received specific advice or support to manage their own health and their carer role [11]. Utilising a dyad-focused approach has been recommended as one approach to improving the assessment and management of carer needs together with those of the person receiving care, although substantial challenges have been reported in terms of implementing this type of approach [67]. Current health and service system constraints, including patient-focused funding models and privacy issues, limit the inclusion of needs assessment and management of older carers of older people [67].

A strength of the approach to developing this novel service is the multiple data sources used in determining the preferred model for the CHWS, that included surveys of older carers of older people [11], interviews with carers and health professionals involved with geriatric out-patient services, information from the only similar service identified internationally (in Sao Paulo, Brazil) [26], and a co-design process of key stakeholders to ensure suitability for the Australian context [28].

The comprehensive mixed methods evaluation will provide valuable effectiveness and implementation outcomes to guide future policy and planning, and potential upscaling of the service model elsewhere nationally and internationally.

## Supporting information

S1 Checklist The SPIRIT 2013 Checklist: Recommended items to address in a clinical trial protocol and related documents. (DOC)

S2 Checklist The TIDieR (Template for Intervention Description and Replication) checklist: Information to include when describing an intervention and the location of the information. (DOCX)
